# Volume fusion of CT images to measure femoral symmetricity

**DOI:** 10.1007/s00276-019-02389-3

**Published:** 2019-11-28

**Authors:** Peyman Bakhshayesh, Olof Sandberg, Vishal Kumar, Adam Ali, Anders Enocson

**Affiliations:** 1 Department of Surgery and Cancer, Imperial College Healthcare, London, UK; 2 Sectra AB, Linköping, Sweden; 3 Imperial College Healthcare, London, UK; 4 Department of Molecular Medicine and Surgery, Karolinska Institute, Stockholm, Sweden

**Keywords:** Image fusion, Surface fusion, CT scan, Human anatomy, Surgical planning

## Abstract

**Purpose:**

Pre-operative planning is widely used in orthopaedic surgery. In case of trauma with fracture or previous injury with malunion, the contralateral extremity is used as a surrogate for planning with an assumption of symmetry between sides. The aim of this study was to investigate femoral symmetricity in human adults.

**Methods:**

Ten randomly selected lower extremity computerized tomography (CT) images were analyzed for femur symmetry using 3D Trauma and CT motion analysis (CTMA). Mirrored images of the left femur were created using the right as a template. The 3D images from each side were merged, and translational and rotational differences reported.

**Results:**

There were no statistically significant differences between mirrored images of the left and right femurs. Differences in rotation and translation of bony segmentation showed a greater variation in internal and external rotation of the distal femur (CI − 0.7° to 4.9°) compared to varus/valgus (CI − 1.3° to 0.8°) or flexion/extension (CI − 0.5° to 0.6°), though none of these differences were significant.

**Conclusion:**

The left and right femurs of healthy adults are symmetrical. Pre-operative templating relying on the contralateral healthy femur is encouraged.

## Introduction

Pre-operative planning is an invaluable tool for orthopaedic surgeons, improving their ability to achieve desired implant positioning. In trauma cases with fractures or previous injuries with malunion, the desired template is not available. The contralateral extremity represents an easily available surrogate in pre-operative planning for lower limb surgery, especially amongst major trauma victims who routinely undergo computed tomography (CT) scans in which both limbs are imaged. However, controversy exists regarding the optimal technique for establishing frames of reference for templating [4]. Whilst some authors report that adult human femurs are symmetrical, others have found an asymmetry that has clear implications for the ability to use the contralateral side as Refs. [4, 5, 9, 14].

Computed tomography scan slices of the extremities are mainly available in Digital Imaging and Communications in Medicine (DICOM) format. The creation of 3D images is then performed by converting these DICOM images to 3D stereolithographic (STL) models [2]. A number of techniques exist to mirror 3D images from the contralateral side to the side of surgical interest with high accuracy for translation, but when it comes to rotational differences, up-to-date literature is lacking [3]. Also, there is a lack of published data investigating symmetricity of the extremities using DICOM images, the fidelity of conversion of DICOM images to STL files and the mirroring of STL files before a 3D image is created. Furthermore, in volume fusion, an automated reporting system would be preferable compared to the standard manual calibration of the images which is subject to bias [1].

Olivecrona et al. recently demonstrated how a combination of 3D Trauma and CT Motion Analysis (CTMA) could be used to study hip prosthetic loosening [7]. Other groups have used the combination of techniques to study fusions after spinal surgery [11, 12]. In a pelvic model, we recently showed that the technique has a precision to report translations with ± 0.2 mm, and angular changes with ± 0.2° [1].

The aim of the current study was to assess the symmetricity of femurs in human adults.

## Materials and methods

Institutional approval from the Imperial College Healthcare, London, United Kingdom, was retrieved. The hospital’s Picture Archiving and Communication System (PACS) was accessed. In patients with no injured femurs, we randomly selected ten consecutive lower extremities CT images performed for major trauma victims from January to December 2018.

Contrast-enhanced CTA scans were performed using a 256-slice Philips Brilliance CT scanner (Koninklijke Philips N.V., Amsterdam, The Netherlands). Gantry was AirGlide, Aperture 700 mm, Focus-isocenter distance 570 mm, and Focus-detector distance 1040 mm. A rotation time of 0.27 s, and Collimation of 2 × 128 × 0.625 mm was used. A field of view (FOV) 200–500 mm and matrix 512 was used. As contrast medium, 70-mL volumes of Omnipaque (General Electric Healthcare, Chicago, IL, USA) were administered intravenously. The iDose4 Premium Package Filter was used. The average tube voltage used was 100 kV, tube current 89–134 mAs, and Dose 520–920 mGy cm.

Images were downloaded as DICOM files, and all files were anonymized, coded, and transferred to a research server. Images were reconstructed in 3D using a 3D Trauma package (Sectra, Linköping, Sweden) creating STL files. Right and left femurs were segmented. The left femur was mirrored using available applications in the 3D Trauma package.

**Fig. 1:**
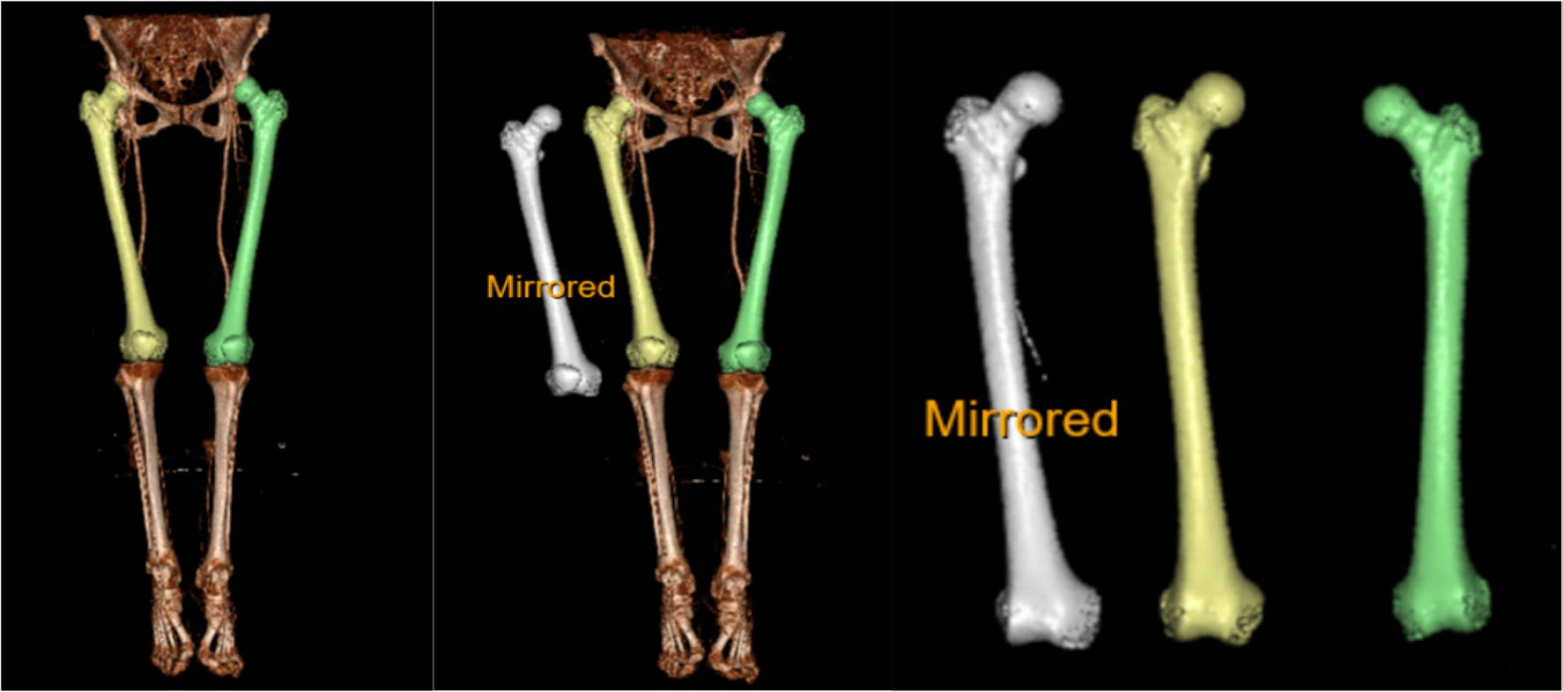
The yellow-colored femur is the original right femur. The white femur is a mirror of the original left femur (green). The comparison was done between the original right and the mirrored left femur

**Fig. 2:**
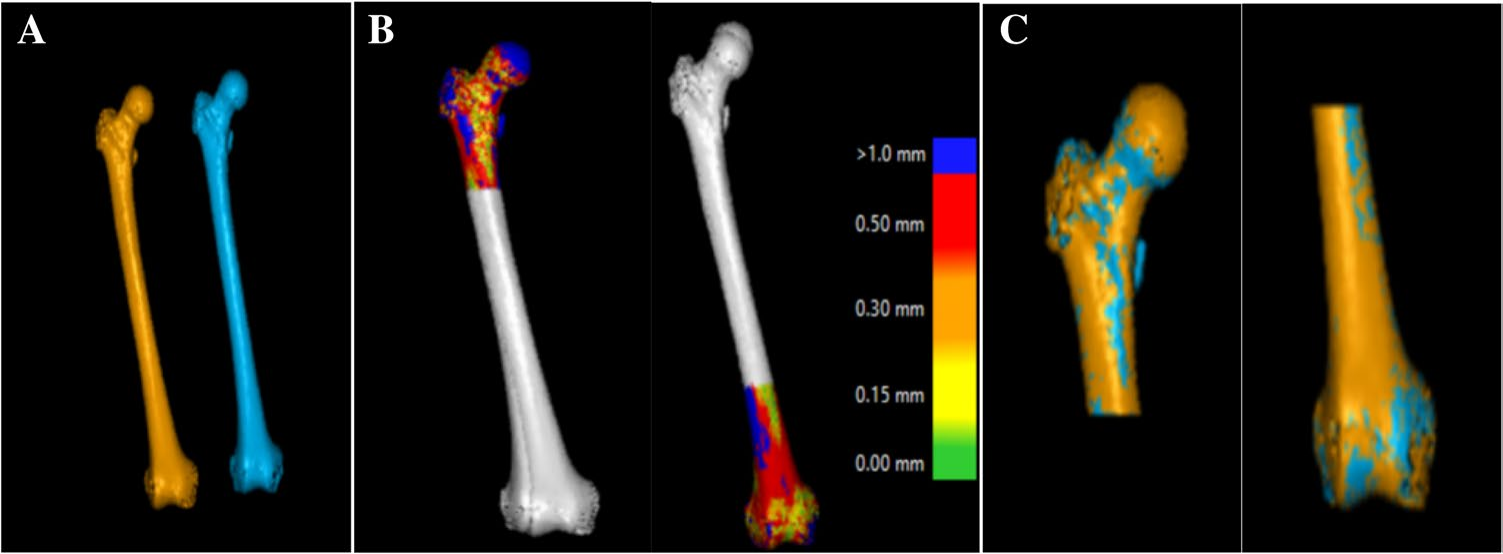
Stages of volume fusion of the proximal and distal femur from (**a**) the original right femur and the mirrored left femur. **b** The color scale indicates how closely the software could place the object of interest in the two CT stacks for the proximal part and the distal part, respectively. **c** The proximal femur is assumed as the static portion (reference) to report rotation and translation of the distal femur

Images of the right femur and mirrored images of left femur were saved (Fig. 1). The software uses two parameters to find the optimal segmentation; first, the user indicates what is the pelvis and what is the femur by clicking on their respective surface in the software. Second, the HU values are used in combination with these clicks to find where one bone ends and another starts (i.e., optimal segmentation). Analyses of images were done using CTMA which is a software that very precisely can find the relative movement of an object between two different CT stacks. This is done by first in both CT stacks randomly spreading up to 100,000 measurement points on the surface of the object of interest. Thereafter, the software rotates and translates the object in the second CT stack to match that in the first CT stack as closely as possible (Fig. 2). This is done by minimizing the distance between the two groups of points. Since the used surfaces are much larger than any artifact areas, this means that artifacts have limited impact on the matching process. The process is done first for a reference object with which the frame of reference is created. Thereafter, the movement of the object of interest is measured in the same way. The process has been previously described in greater detail [1].

Based on our previous experience using this software, 10,000 points with a mean distance difference between meshes of 0.5 mm or less was chosen [1]. No smoothing was used in the CTMA software.

The proximal part of the right femurs including the head of the femur, the greater trochanter, and the lesser trochanter of each STL created 3D volume were merged with the mirrored contralateral side (Fig. 2). These merged images were saved as static, or nonmoving parts, and were used as reference volumes. Furthermore, the distal part of the femurs including both condyles, the inter-condylar sulcus, and the supra-condylar area were merged.

The CTMA package offers translational and rotational changes in three different Euler axes (*X*, *Y,* and *Z*) [7, 8]. These axes and the rotations were defined as per DICOM standard; axis *X* from the patient’s left to the right, axis *Y* from the patient´s front to back, and axis *Z* from the patient’s feet to head. Positive rotation was defined as clockwise when looking along the positive axis direction. Translational changes were reported either for the entire volume of an object based on Centre Of Mass (COM) of the 10,000 points spread on the surface. This COM was similar but not identical to the mathematical centre of the geometric volume on which the points were spread out. Rotation was reported for the entire geometrical volume.

Statistics: accuracy was analyzed as per root-mean-square error (RMSE) with mean, median, and 95% confidence interval (CI) of the mean [10]. Shapiro–Wilk and Kolmogorov–Smirnov tests were used to test the distribution of normality. Statistical analysis was performed using IBM SPSS Statistics version 25 for Windows. A *p* value < 0.05 was considered statistically significant.

## Results

**Fig. 3:**
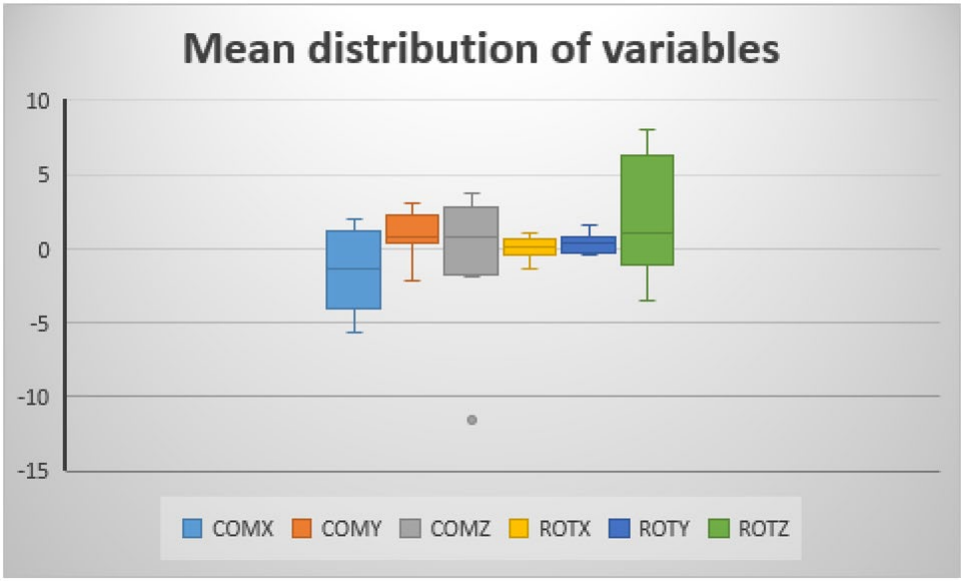
The confidence interval (CI) of all measurements. *COM* centre of mass translational difference, *ROT* rotational difference. *X*, *Y,* and *Z* represent the three Euler’s axis for COM and ROT, respectively

The mean age of the study population was 54 ± 20 years. There were six males and four females. Eight of the cases were of white British origin, one black African, and one from the Middle East. Differences in rotation and translation with error bars for translational measurement differences in *X*, *Y,* and *Z*-axes of COMs are presented in Table 1. We observed a greater variation in internal and external rotation of the distal femur (CI −0.7° to 4.9°) compared to varus/valgus (CI −1.3° to 0.8°) or flexion/extension (CI − 0.5° to 0.6°). None of these differences were statistically significant. Tests of normality of distribution of the variables are presented in Table 2. Apart from COMZ, all other variables were normally distributed. The CI of all measurements crossed zero (Table 1, Fig. 3). Differences between the right femur and the mirrored images of the left femur for any patient greater than 3.6 mm or 5° could be excluded with a 95% confidence.

**Table 1:** All measurements

Subject	COMX mm Median: − 1.3 Mean: − 1.5 95% CI Mean: − 3.5 to 0.4	COMY mm Median: 0.8 Mean: 0.9 95% CI Mean: − 0.1 to 2.0	COMZ mm Median: 0.8 Mean: − 0.1 95% CI Mean: − 3.2 to 3.1	ROTX degrees Median: 0.1 Mean: 0.03 95% CI Mean: − 0.5 to 0.6	ROTY degrees Median: 0.4 Mean: 0.3 95% CI Mean: − 1.3 to 0.8	ROTZ degrees Median: 1.0 Mean: 2.1 95% CI Mean: − 0.7 to 4.9
1	− 3.832	2.235	0.768	0.087	0.947	6.004
2	− 1.899	1.219	− 1.886	− .044	0.723	4.344
3	− 2.909	2.336	− 11.564	0.516	1.545	1.483
4	− 4.934	0.031	3.732	− 1.396	0.724	6.969
5	1.047	− 2.125	0.917	0.012	− 0.181	− 3.446
6	− 5.640	0.653	3.666	− 1.348	0.709	8.011
7	− 0.786	0.555	2.534	0.393	0.112	− 1.858
8	− 0.250	3.135	2.205	0.846	− 0.180	0.597
9	1.964	0.907	0.510	1.096	− 0.432	− 0.868
10	1.874	0.738	− 1.643	0.214	− 0.466	− 0.211

*COM* centre of mass translational difference, *ROT* rotational difference, *CI* confidence interval. *X*, *Y,* and *Z* represent the three Euler’s axis for COM and ROT, respectively

**Table 2:** Test of normal distribution of the variables

	Tests of normality					
	Kolmogorov–Smirnov			Shapiro–Wilk		
	Statistic	*df*	Sig	Statistic	*df*	Sig
COMX	0.126	10	0.200	0.940	10	0.548
COMY	0.188	10	0.200	0.934	10	0.484
COMZ	0.252	10	0.071	0.761	10	0.005
ROTX	0.261	10	0.053	0.878	10	0.125
ROTY	0.203	10	0.200	0.917	10	0.331
ROTZ	0.162	10	0.200	0.938	10	0.528

*X*, *Y,* and *Z* represent the three Euler’s axis for COM and ROT, respectively

*COM* centre of mass translational difference, *ROT* rotational difference, *df* degree of freedom, *Sig* significance

## Discussion

The main finding of our study was that the left and right femurs of healthy adults were highly symmetrical. This was found using a novel image fusion technique. Based on the findings of this study, it is, therefore, appropriate to perform pre-operative templating using the unaffected side in orthopaedic surgery.

Previous studies have shown asymmetry of proximal femurs [4, 5]. Eckhoff et al. used the SOMA (Stryker Orthopaedic Modeling and Analytics) system to create 3D images of 361 femurs. The authors utilized a digitally available measurement technique which demands operator-based drawing of the lines and manual/digital measurements. Unfortunately, the authors used a single observer at each occasion. Whilst the authors reported a mean difference of 7 degrees between femurs, the reproducibility of their pre-defined points to measure translation or angular changes is, therefore, debatable [4].

In our presented technique, we do not rely on user-defined anatomical landmarks which could be subject to bias [6]. Introduction of volume fusion together with an automated technique has simplified reporting differences in angulation and translation substantially in other areas, and our study lends support to its use for pre-operative templating involving the femur [1, 7, 11–13].

One limitation of our study was the number of subjects as we used only ten cases in our study. However, with the high accuracy associated with the 3D-CT and CTMA techniques 3D, we are confident that the number of patients was sufficient to be able to describe femur symmetricity accurate enough to be used in a clinical situation. In addition, as our data showed normal distribution, we believe that adding more patients to our study would only make our confidence interval narrower and the final result and conclusion would be the same. Another limitation of the study is that in patients with leg-length inequality or other abnormal anatomy, the use of a mirrored femur for templating can be of limited use.

## Conclusion

Using a volume fusion technique to superimpose the affected and contralateral limb, we have demonstrated symmetricity of the femurs that lends support to the use of the contralateral side for pre-operative templating. Volume fusion is a promising technique within orthopaedics and may greatly simplify the ability to plan surgery.
